# Facilitation of neocortical presynaptic terminal development by NMDA receptor activation

**DOI:** 10.1186/1749-8104-7-8

**Published:** 2012-02-16

**Authors:** Michael P Sceniak, Corbett T Berry, Shasta L Sabo

**Affiliations:** 1 Departments of Pharmacology and Neurosciences, Case Western Reserve University School of Medicine, 10900 Euclid Avenue, Cleveland, OH 44106, USA

## Abstract

**Background:**

Neocortical circuits are established through the formation of synapses between cortical neurons, but the molecular mechanisms of synapse formation are only beginning to be understood. The mechanisms that control synaptic vesicle (SV) and active zone (AZ) protein assembly at developing presynaptic terminals have not yet been defined. Similarly, the role of glutamate receptor activation in control of presynaptic development remains unclear.

**Results:**

Here, we use confocal imaging to demonstrate that NMDA receptor (NMDAR) activation regulates accumulation of multiple SV and AZ proteins at nascent presynaptic terminals of visual cortical neurons. NMDAR-dependent regulation of presynaptic assembly occurs even at synapses that lack postsynaptic NMDARs. We also provide evidence that this control of presynaptic terminal development is independent of glia.

**Conclusions:**

Based on these data, we propose a novel NMDAR-dependent mechanism for control of presynaptic terminal development in excitatory neocortical neurons. Control of presynaptic development by NMDARs could ultimately contribute to activity-dependent development of cortical receptive fields.

## Background

Synapse formation is a key step in the assembly of the neural circuits that control perception and behavior. In addition, impaired synapse formation may contribute to abnormal development and pathogenesis of neurodevelopmental disorders. Recent genetic and functional studies have linked synapse development to autism spectrum disorders, cognitive impairment, epilepsy, schizophrenia and depression [1,2].

During glutamatergic synapse formation in the central nervous system, contact between an axon and a dendrite induces a cascade of events, ultimately resulting in formation of a presynaptic terminal and postsynaptic density at the site of contact. As an integral part of this process, the proteins required for regulated transmitter release must be accumulated at the site of axo-dendritic contact. It has been shown that transport vesicles deliver synaptic vesicle (SV) and active zone (AZ) proteins to developing presynaptic terminals [1,3,4]. Formation of the AZ is then thought to be initiated by fusion of AZ protein transport vesicles with the axonal surface [5]. SVs form within the nascent terminal or are acquired from preassembled clusters of SVs that are mobile within axons [6-9]. As a bouton continues to develop, the number of SVs increases and the AZ expands, requiring continual recruitment of SV and AZ proteins [10]. The molecular signals that control accumulation of SV and AZ proteins at nascent terminals remain incompletely understood.

Although, the initial induction of synapse formation occurs through trans-synaptic molecular interactions [1,11] even in the absence of neurotransmitter release and glutamate receptor activation, synaptic activity and glutamate can modulate synaptic development [12-14]. For example, synaptic activation of NMDA receptors (NMDARs) is sufficient to induce growth of new dendritic spines and synapses [15-19]. In addition, knockdown or knockout of postsynaptic NMDARs alters the density and structural dynamics of dendritic spines and the accumulation of postsynaptic scaffolding molecules at spines [20-23], although changes in density may be at least partially a result of altered dendrite growth and branching [20,24]. Finally, activation of NMDARs regulates development of AMPA receptor currents and clustering of NMDARs [25-27].

Although most previous studies have focused on the role of glutamate receptors during postsynaptic development, it is likely that glutamate receptors also control presynaptic development since presynaptic and postsynaptic morphology and function are correlated [28,29]. For example, during early stages of circuit development, synapses with high accumulation of postsynaptic markers have high accumulation of presynaptic markers [28]. Regulation of presynaptic development by glutamate receptors could occur through direct effects on presynaptic terminals, possibly via presynaptic NMDARs [30,31], via activation of postsynaptic receptors followed by retrograde signaling from postsynaptic dendrites to presynaptic terminals [32], or by cell-autonomous transneuronal signaling from postsynaptic receptors to presynaptic terminals [33-38].

Recent reports suggest that synaptic activity regulates presynaptic terminal development. For example, Munc18-1 knockouts that lack transmitter release have decreased synapse density, synapses with docked SVs, and number of SVs [39]. In another study, the total levels of several SV proteins were decreased in the hippocampus from mice with diminished glutamatergic transmission due to knockout of VGlut1 [40]. Finally, in *Xenopus *optic tectum, bath application with the use-dependent NMDAR inhibitor MK-801 leads to a decrease in the percentage of presynaptic volume occupied by SVs [10].

Here, we used confocal fluorescence imaging to investigate the role of NMDAR activation at developing presynaptic terminals. We find that activation of NMDARs controls accumulation of both SV and AZ proteins at nascent presynaptic terminals. This regulation occurs even in the absence of retrograde signaling from postsynaptic NMDARs or glia. Based on these observations, we suggest a model for synapse development in which NMDARs directly regulate presynaptic terminal assembly. This could provide a feed-back mechanism capable of promoting development of active presynaptic terminals, even in the absence of postsynaptic activity.

## Results

### NMDA receptor activation regulates accumulation of synaptic vesicle proteins during synapse formation

**Figure 1:**
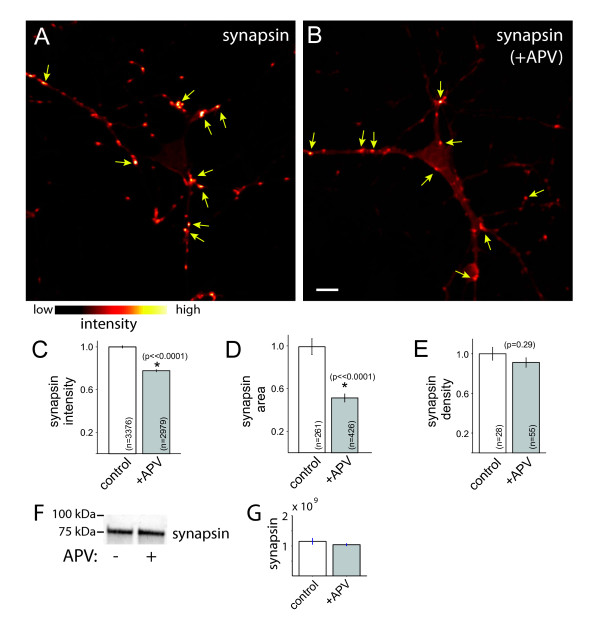
**NMDAR activation controls accumulation of synapsin at developing excitatory presynaptic terminals**. **(A, B) **Immunofluorescence images of synapsin in a control neuron (A) and a neuron treated with APV to block NMDAR activation (B). Fluorescence intensity is pseudo-colored according to the indicated scale. Scale bar: 10 μm. **(C) **APV treatment decreases accumulation of synapsin at developing presynaptic terminals. To ensure that presynaptic puncta were synaptic, analysis was limited to synapsin puncta that overlapped with PSD95. **(D) **APV similarly decreased the apparent size of synapsin puncta. **(E) **APV did not significantly decrease the density of synapsin puncta. Data are presented as mean ± standard error of the mean, normalized to the control mean, and the number of puncta (C-D) and images (E) measured are indicated on each bar. *, significant difference (P-values are indicated in the figure). **(F) **Immunoblot demonstrating that overall levels of synapsin were unchanged by APV treatment. **(G) **Quantification of synapsin integrated density from immunoblotting. The small difference in intensity was not significant (*P *= 0.28).

One of the earliest steps of presynaptic development is obligatory recruitment of SV proteins to nascent synapses. Therefore, to assess whether activation of NMDA receptors plays a role in excitatory presynaptic terminal development, neurons were treated with APV (DL-2-amino-5-phosphonopentanoic acid) for 24 to 48 h then fixed and immunolabeled with antibodies against the SV-associated protein synapsin and imaged using confocal microscopy (Figure 1A,B). To focus on early stages of synapse assembly, neurons were treated at the beginning of excitatory synapse formation, at 6 to 7 days *in vitro *(DIV), then synapse development was assayed at 8 DIV. Simultaneous immunolabeling for tau-1 was used to identify axons, and PSD95 labeling verified that synapsin was associated with synapses. Synapsin puncta were identified using an automated image analysis algorithm (see Materials and methods) and their fluorescence intensities quantified.

NMDAR blockade with APV resulted in a significant decrease in the mean synapsin immunofluorescence intensity (at puncta that co-localized with the postsynaptic marker PSD95; Figure 1A-C; *P *< 0.00001; n = 3,376 (control) and 2,979 (+APV) puncta). Similarly, the mean apparent size of synapsin puncta was decreased by APV treatment (Figure 1D; *P *< 0.00001). As illustrated in the immunoblot shown in Figure 1F, overall synapsin levels were not significantly decreased by APV treatment (*P *= 0.28, n = 4 lanes for each condition from 2 experiments, integrated density of control = 1.15 ± 0.09 × 10^9 ^and APV = 1.04 ± 0.035 × 10^9 ^(Figure 1G)). Consistent with this, levels of synapsin immunofluorescence measured at the soma or overall levels in large fields of view were not significantly changed by NMDAR inhibition (somas: *P *= 0.1, n = 18 neurons each for control (876 ± 154 arbitrary units) and APV-treatment (508 ± 91 arbitrary units); large fields of view: *P *= 0.86, n = 18 images for each condition, 181 ± 25 and 280 ± 102 arbitrary units for control and APV-treated, respectively). In addition, the density of synapsin-positive presynaptic terminals was not significantly altered by NMDAR inhibition (Figure 1E; *P *= 0.29, n = 28 and 55 images of control and APV-treated axons, respectively).

**Figure 2:**
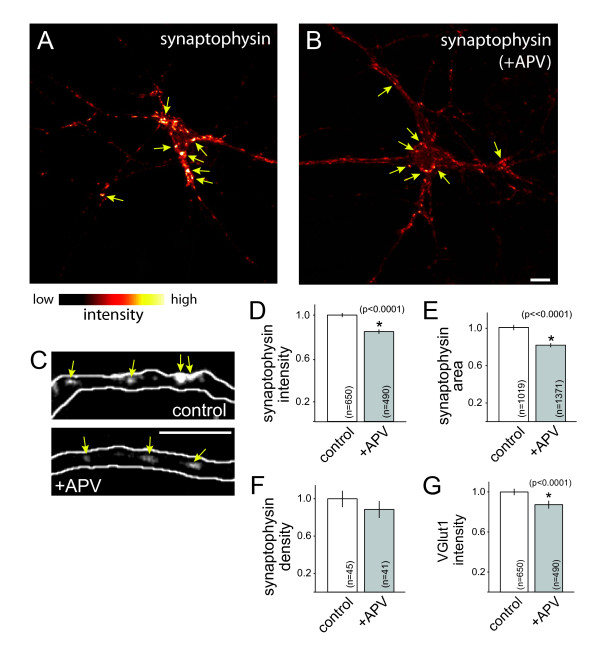
**NMDAR activation controls accumulation of multiple synaptic vesicle proteins at developing excitatory presynaptic terminals**. **(A, B) **Immunofluorescence images of synaptophysin in a control neuron (A) and a neuron treated with APV to block NMDA receptors (B). Fluorescence intensity is pseudo-colored according to the indicated scale. **(C) **Higher magnification immunofluorescence images of synaptophysin (arrows) in a GFP-transfected axon (white outline). Left, control neuron; right, neuron treated with APV to block NMDAR activation. Scale bars: 10 μm. **(D, E) **APV treatment decreased accumulation of synaptophysin at developing presynaptic terminals. Both the intensity (D) and apparent sizes (E) of synaptophysin puncta were decreased upon APV exposure. **(F) **APV did not change presynaptic terminal density. **(G) **Blockade of NMDA receptors with APV decreased the expression of VGlut1 at terminals. Data are presented as normalized mean ± standard error of the mean, and the number of puncta (D, E, G) and axons (F) measured are indicated on each bar. *, significant difference (P-values are indicated in the figure).

To determine whether the observed effects correspond to a general change in SV proteins, similar experiments were performed using the integral SV protein, synaptophysin. Synaptophysin puncta were significantly less bright in neurons treated with APV than in control neurons (Figure 2A-D; *P *< 0.0001; n = 650 and 490 puncta from 45 and 41 axons for control and +APV, respectively). The mean area of synaptophysin-positive puncta was also decreased by APV treatment (Figure 2E). These data indicate that NMDAR activation modulates accumulation of synaptophysin at excitatory synapses. When overall levels of synaptophysin immunofluorescence were quantified at neuron somas rather than synapses or in large fields of view, synaptophysin intensity was unchanged by APV treatment (see Figure 2A,B for examples; somas: *P *= 0.1, fluorescence intensity (normalized to control) = 1.00 ± 0.05 and 1.12 ± 0.05 for n = 25 and 23 somas (control and APV-treated, respectively); overall fluorescence: *P *= 0.64, intensity = 1.00 ± 0.04 and 1.04 ± 0.04; for 33 and 34 fields of view for control and +APV, respectively).

In order to quantify the effects of NMDAR blockade on presynaptic terminal density, neurons were transfected with GFP to allow us to trace individual axons and, therefore, quantify the density of synaptophysin puncta in individual axons. Although there was a small decrease in the density of synaptophysin puncta in APV-treated axons when compared to untreated axons, the observed difference was not statistically significant (Figure 2F), consistent with previous observations [26,41,42].

During this stage of development, the vesicular glutamate transporters responsible for loading SVs with glutamate undergo a change, with dramatic up-regulation of VGlut1 expression concomitant with decreased VGlut2 expression [40,43]; therefore, we also tested whether blockade of NMDARs with APV results in a decrease in VGlut1 expression at synapses. When neurons were treated with APV from 5 to 8 DIV, VGlut1 intensity at presynaptic terminals decreased significantly (Figure 2G; *P *< 0.0001; n = 650 and 490 puncta from 45 and 41 axons of control and APV-treated neurons, respectively). Therefore, the level of VGlut1 at synapses is also modulated by activation of NMDARs. These data demonstrate, for the first time, that NMDAR activation regulates accumulation of multiple SV proteins during excitatory presynaptic development.

### NMDAR activation controls accumulation of synaptic vesicle proteins at synapses that lack postsynaptic NMDARs

**Figure 3:**
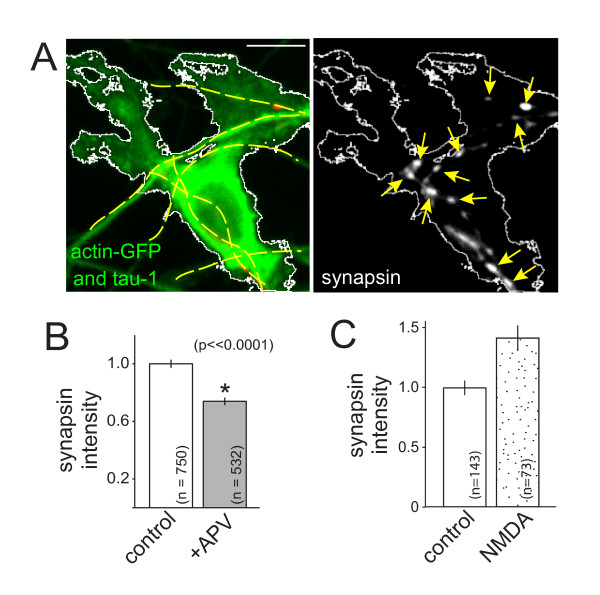
**NMDAR-dependent accumulation of synaptic vesicle proteins at developing presynaptic terminals is independent of postsynaptic NMDARs and bi-directional**. **(A) **Synapsin accumulates at presynaptic terminals induced by contact with neuroligin-expressing HEK-293 cells. The neuroligin-expressing cell is identified by co-expression of actin-GFP (green) and is outlined in both panels (white). Axons were labeled with tau-1 antibodies to identify contacts and are visible in the left panel (green, highlighted with yellow dashed lines). Induced synapsin puncta are indicated by arrows (right panel). The left panel also shows the overlay of the synapsin (red) and PSD95 (blue). PSD95 label does not co-localize with induced synapsin puncta. Scale bar: 10 μm. **(B) **Accumulation of synapsin is decreased by APV at presynaptic terminals that lack postsynaptic partners. **(C) **Direct activation of NMDARs via treatment with NMDA (0.015 mM for 24 h) increased the fluorescence intensity of synapsin puncta (*P *< 0.0001). Data are normalized to the control mean and presented as normalized mean ± standard error of the mean. The numbers of puncta quantified for each condition are indicated on the plots in (B, C). *, significant difference (P-values are indicated in the figure).

Since postsynaptic NMDARs are already present at nascent synapses within minutes to hours of axo-dendritic contact [44], we next tested whether postsynaptic signaling mediates the observed effects of NMDAR activation. To test whether postsynaptic signaling is required, hemi-synapses were formed that contained presynaptic terminals but lacked postsynaptic densities. These hemi-synapses were formed by taking advantage of the well-established observation that contact between an axon and a neuroligin-expressing non-neuronal cell is sufficient to induce presynaptic terminal assembly, even in the absence of dendritic contact [45]. For this assay, HEK-293 cells were transfected with neuroligin. Co-transfection of the HEK-293 cells with actin-GFP allowed visualization of neuroligin-expressing cells, including their lamellipodial extensions. The neuroligin-expressing cells were placed into contact with axons of 6 to 7 DIV neurons, and half of the co-cultures were treated with APV (0.1 mM). After 24 to 48 h, cells were fixed and immunolabeled. Presynaptic puncta induced by neuroligin were identified by overlap with actin-GFP in the contacting HEK-293 cell, tau-1 in the axon and the absence of PSD95, to indicate the absence of a postsynaptic partner (Figure 3A).

Surprisingly, when neurons were treated with APV, the intensities of synapsin puncta associated with neuroligin-expressing cells decreased significantly (Figure 3B; *P *< 0.0001; n = 750 (control) and 532 (+APV) puncta). Importantly, treatment with APV did not significantly alter the expression of neuroligin (*P *> 0.05; n = 63 (control) and 66 (+APV) HEK-293 cells; data not shown). These data demonstrate that postsynaptic receptor activation is not required for NMDAR-dependent modulation of excitatory presynaptic development.

### Direct activation of NMDARs increases synaptic vesicle protein accumulation

To determine whether accumulation of presynaptic proteins can be enhanced by increased NMDAR activation, neurons were treated for 24 h with NMDA, a highly selective NMDAR agonist. Synapsin immunofluorescence was imaged and quantified at presynaptic terminals induced by contact with neuroligin-expressing HEK-293 cells. Direct activation of NMDARs yielded an increase in the intensity of synapsin fluorescence (Figure 3C; *P *< 0.0001; n = 143 (control) and 73 (NMDA-treated) puncta). These data demonstrate that presynaptic protein accumulation is bi-directionally controlled by activation of NMDARs.

### Activation of NMDARs regulates accumulation of active zone proteins at presynaptic terminals

**Figure 4:**
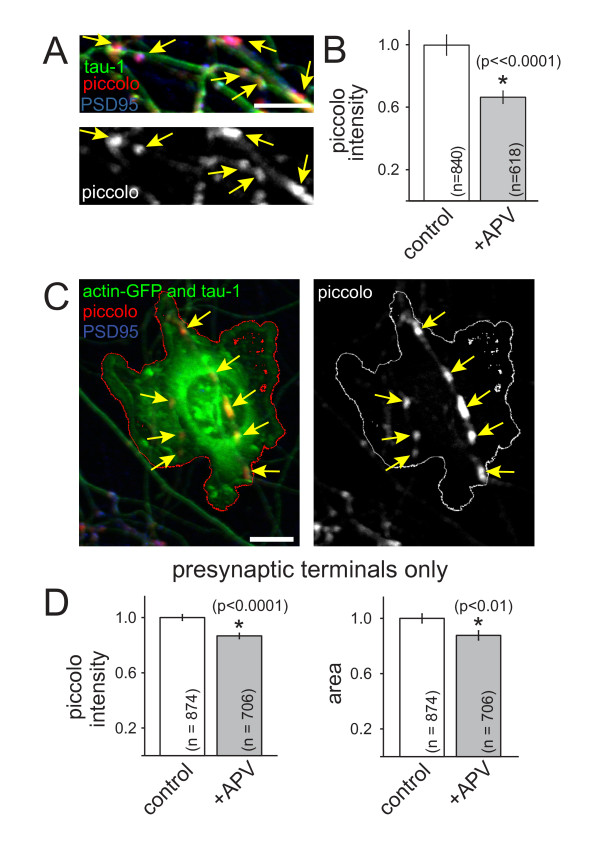
**NMDAR activation controls accumulation of active zone proteins at developing excitatory presynaptic terminals**. **(A) **Fluorescence images of the AZ protein piccolo (red, left panel; white, right panel) in young axons (tau-1, green). Piccolo was at synapses since it co-localized with PSD95 (blue). Scale bars: 10 μm. **(B) **Treatment with APV decreased accumulation of piccolo at presynaptic terminals that co-localized with PSD95. **(C) **Piccolo (arrows; red, left panel; white, right panel) is recruited to presynaptic terminals induced by contact with neuroligin and actin-EGFP-expressing HEK-293 cells (green and outline). These piccolo puncta did not co-localize with PSD95 (blue). Axons were labeled with tau-1 antibodies (green) to identify contacts. **(D) **APV decreased accumulation of piccolo at presynaptic terminals that lack postsynaptic partners (left). APV also decreased the apparent sizes of piccolo puncta (right). Data are presented as normalized mean ± standard error of the mean, and the number of puncta quantified are indicated on each bar. *, significant difference (P-values are indicated in the figure).

SV and AZ proteins are delivered to developing synapses via distinct precursor vesicles [1]. Therefore, we next tested whether the observed changes in SV protein accumulation were due to either a specific change in SVs or a more general change in presynaptic terminal development. To distinguish between these possibilities, accumulation of the AZ matrix protein piccolo was measured in similar experiments (Figure 4A). When neurons were treated with APV for 24 to 48 h at 6 to 7 DIV, the mean intensity of piccolo puncta was significantly decreased in APV-treated neurons (*P *< 0.0001; n = 1,103 and 807 puncta for control and +APV, respectively). When analysis was limited to piccolo puncta that exhibited colocalization with PSD95, similar decreases in piccolo intensity were observed (Figure 4B; *P *< 0.00001; n = 840 and 618 puncta for control and +APV, respectively). These data demonstrate that NMDAR activation controls accumulation of both AZ and SV proteins at developing synapses.

Next, we determined whether NMDAR-dependent regulation of AZ development is also independent of postsynaptic signaling. To do so, axons were contacted with HEK-293 cells expressing neuroligin and actin-GFP to generate synapses that lack postsynaptic NMDARs, as described above for SV proteins (Figure 4C). The intensity of piccolo was significantly decreased at presynaptic terminals that were induced by contact with neuroligin (Figure 4D; *P *< 0.0001; n = 874 (control) and 706 (+APV) puncta). The mean size of piccolo puncta was also decreased at these presynaptic terminals (Figure 4D; *P *< 0.01, n = 874 (control) and 706 (+APV) puncta). These results indicate that NMDARs control accumulation of AZ proteins during excitatory presynaptic terminal development, even in the absence of retrograde signals from postsynaptic partners. Together, our data are consistent with the hypothesis that NMDAR activation regulates the assembly of multiple classes of synaptic proteins at developing excitatory presynaptic terminals.

### Blockade of non-NMDA glutamate receptors but not network activity decreases presynaptic protein accumulation

**Figure 5:**
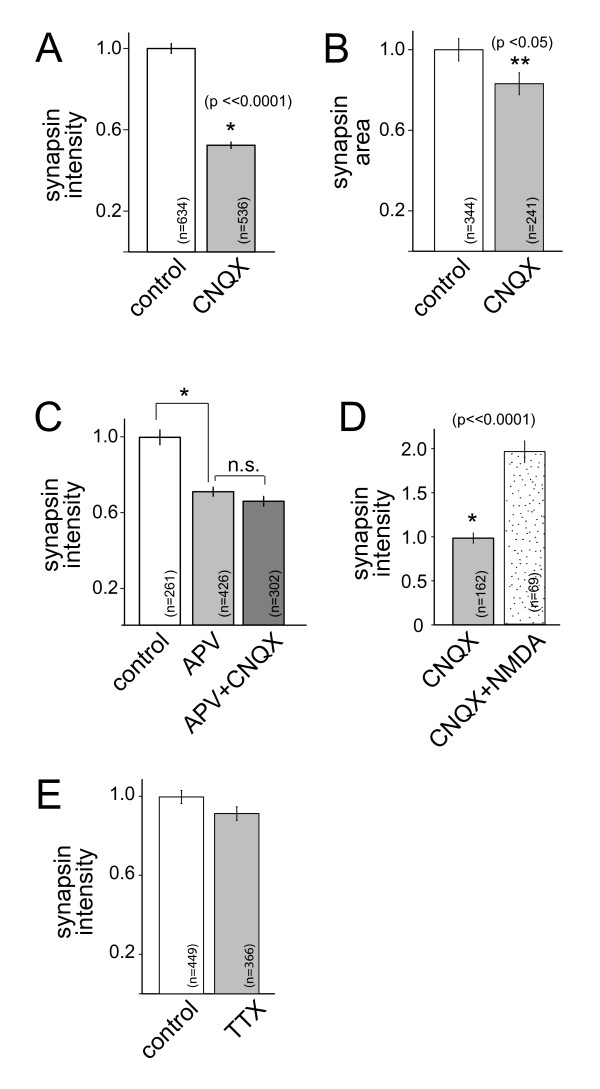
**Accumulation of synaptic vesicle proteins is decreased by blockade of non-NMDA glutamate receptors but not action potential activity in the entire network**. **(A) **Treatment of neurons with the AMPA/kainate receptor inhibitor CNQX resulted in decreased synapsin immunofluorescence intensity, similar to that observed with APV. **(B) **The apparent size of synapsin puncta was also decreased by CNQX exposure. **(C) **The effects of APV and CNQX on synapsin accumulation were not additive. Although APV application resulted in a significant decrease in synapsin intensity (*P *<< 0.0001), addition of APV plus CNQX yielded a decrease that is not quantitatively different from the decrease induced by APV application alone (*P *= 0.15). Similarly, the effect of APV plus CNQX application on the apparent size of synapsin puncta was also not different from that observed with treatment with APV alone (data not shown, *P *= 0.27). **(D) **Treatment of neurons with NMDA reversed the effects of CNQX on the intensity of synapsin puncta. **(E) **Treatment of neurons with TTX did not significantly decrease the intensity of synapsin puncta (*P *= 0.079). Data are presented as the mean ± standard error of the mean and are normalized to control values. The numbers of puncta measured for each condition are indicated on the plots. N.s., not significant. *, highly significant difference and **, significant difference (P-values are indicated in the figure).

In addition to NMDA receptors, cortical neurons express AMPA (α-amino-3-hydroxy-5-methyl-4-isoxazolepropionic acid) and kainate receptors. To determine whether the effects of NMDAR activation were limited to NMDARs, neurons were treated with the AMPA/kainate receptor blocker CNQX (6-cyano-7-nitroquinoxaline-2,3-dione; 0.025 mM) as described above for APV. Then synapsin immunofluorescence was imaged and quantified at presynaptic terminals induced by contact with HEK-293 cells expressing neuroligin. Upon treatment with CNQX, synapsin immunofluorescence was decreased (Figure 5A; n = 634 (control) and 536 (+CNQX) puncta). Similar to what was observed upon NMDAR blockade, the apparent size of synapsin puncta was also decreased (Figure 5B; n = 344 (control) and 241 (+CNQX) puncta) when non-NMDA glutamate receptors were inhibited. When neurons were treated in parallel with APV (0.1 mM), CNQX (0.025 mM), or APV plus CNQX (0.1 and 0.025 mM, respectively), all three treatments caused similar decreases in synapsin fluorescence (Figure 5C; n = 261 (untreated), 426 (APV-treated), and 302 (APV+CNQX-treated) puncta). APV and CNQX treatment were not additive. In addition, the effects of CNQX on synapsin immunofluorescence could be reversed by treatment with NMDA (0.015 mM; Figure 5D; n = 162 (+CNQX) and 69 (+CNQX and NMDA) puncta). Interestingly, treatment with the sodium channel blocker tetrodotoxin (TTX; 0.001 mM) did not alter synapsin accumulation at presynaptic terminals (Figure 5E; n = 449 (control) and 366 (TTX-treated) puncta), suggesting that the observed effects of glutamate receptor activity are not a result of decreased action potential-evoked activity.

### NMDAR-dependent control of presynaptic protein accumulation is independent of glial glutamate

**Figure 6:**
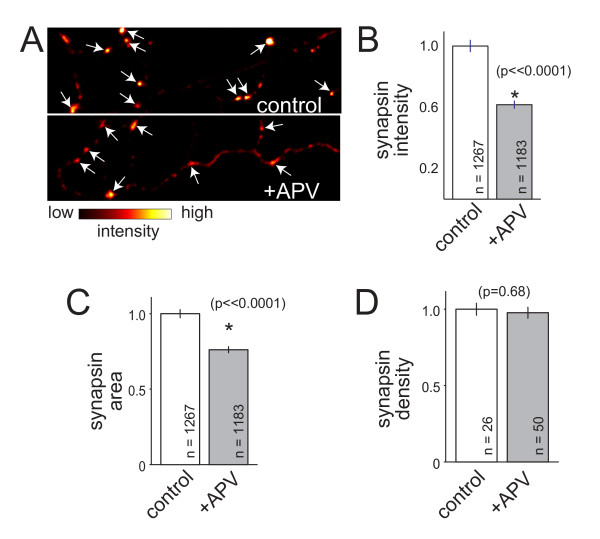
**NMDAR-dependent accumulation of synaptic vesicle proteins does not depend on interactions with glia**. **(A) **Images of synapsin immunofluorescence (arrows) at presynaptic terminals of neurons grown in the absence of glia. Images are pseudo-colored such that the highest intensities are white while the lowest are black, as indicated by the intensity scale below the images. Top, control neuron; bottom, APV-treated neuron. **(B, C) **Synapsin intensity and apparent size at presynaptic terminals were significantly reduced when neurons were treated with APV even when glia were not present. **(E) **Similar to what was observed in cultures grown in contact with glia, the density of synapsin-positive presynaptic puncta remained unchanged. Data are presented as normalized mean ± standard error of the mean, and the number of puncta measured are indicated on the plot. *, significant difference (P-values are indicated in the figure).

Previous reports have shown that glia can release glutamate [46-49]. Therefore, the effects of NMDAR activation on presynaptic development could be due to presynaptic terminals sensing glutamate released from glia. To test this, neurons were cultured without either a glial monolayer or glial conditioning of medium then treated with APV and immunolabeled to quantify SV protein accumulation, as described above. In these cultures, inhibition of NMDAR activation with APV resulted in a decrease in the intensity of synapsin labeling at presynaptic terminals (Figure 6A,B; *P *< 0.00001; n = 1,267 (control) and 1,183 (+APV) presynaptic terminals). The average apparent size of synapsin puncta was also smaller upon inhibition of NMDARs (Figure 6C; *P *< 0.00001; n = 1,267 (control) and 1,183 (+APV) puncta). Similar to what was observed when neurons were grown on top of glia, the density of synapsin puncta was unchanged by APV (Figure 6D; *P *= 0.68, n = 26 and 50 images of control and +APV axons, respectively). Non-neuronal cells were only present in these cultures at a density of approximately 3.7 × 10^-12^/mm^2^, and only fields of view that lacked any glia were imaged for quantification of synapsin; therefore, glia could not have released glutamate near the quantified synapses. While it remains formally possible that the few glia present in the cultures could have contributed to ambient glutamate levels, on average les than 900 glia were present for each 1 ml of culture medium, making it unlikely that glutamate released by these glia was a major source for NMDAR activation. These data suggest that glutamate released by glia is not required for activation of NMDARs during development of presynaptic terminals.

## Discussion

The mechanisms that control accumulation of SV and AZ proteins at developing presynaptic terminals remain poorly understood. Here, we tested whether NMDAR activity plays an important role in presynaptic terminal development. We showed that activation of NMDA receptors regulates accumulation of both SV and AZ proteins at developing synapses. By imaging contacts between axons and HEK-293 cells that express the synaptogenic protein neuroligin, we were able to study presynaptic development in the absence of postsynaptic signaling. Using this approach, we demonstrated that activation of NMDARs controls accumulation of both SV and AZ proteins at developing presynaptic terminals, independent of postsynaptic receptor activation. Although it is still possible that postsynaptic NMDAR signaling can also contribute to this process, the magnitude of the NMDAR-dependent change in synaptic protein accumulation at terminals that lack postsynaptic NMDARs was sufficient to explain the effects of blockade of all NMDARs. Importantly, our results using neuroligin-expressing HEK-293 cells also argue against indirect effects of axon and dendrite growth since presynaptic terminals were induced in regions of axon that had already grown and dendrites were not involved. The effects observed here could be due to either changes in recruitment of presynaptic proteins to presynaptic terminals or changes in transcription, translation or stability of synaptic proteins. We did not observe overall changes in the levels of synapsin or synaptophysin, but contributions from local changes in synthesis or stability are still possible.

In contrast to a plethora of studies on the role of activity in control of synapse density and postsynaptic receptor recruitment [10,12,23], few studies have focused on control of important aspects of presynaptic development, such as the accumulation of synaptic proteins at presynaptic terminals. Immunoblotting of VGlut1 knockout mouse hippocampus showed that overall levels of SV proteins were decreased [40]. Our data show a similar reduction of SV proteins in neocortical neurons and extend these observations to demonstrate that changes in SV protein expression occur at the level of individual synapses and are accompanied by decreases in AZ proteins.

**Figure 7:**
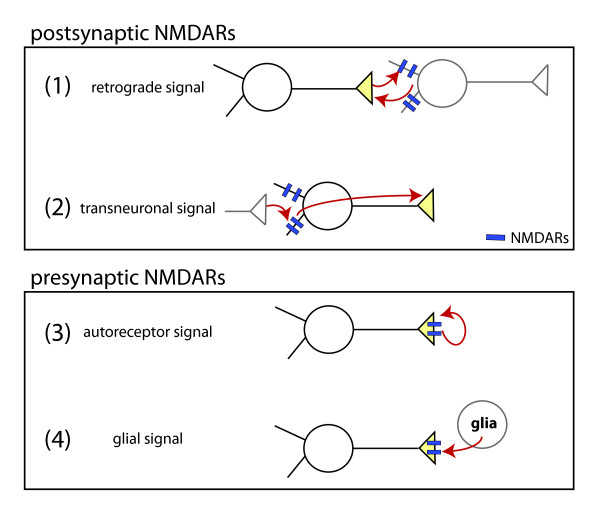
**Models of presynaptic terminal development by activation of NMDARs**. NMDAR activation by glutamate could regulate presynaptic development via at least four mechanisms: (1) glutamate released from presynaptic terminals could activate postsynaptic NMDARs, then postsynaptic neurons could release a retrograde signal that controls presynaptic development; (2) activation of postsynaptic NMDARs could induce transmission of a transneuronal signal, regulating presynaptic development in the activated neuron; (3) glutamate released from presynaptic terminals could activate presynaptic autoreceptors to cell-autonomously regulate presynaptic development; (4) glia could release glutamate to activate NMDARs and regulate presynaptic development. Mechanisms 1 and 2 are mediated by activation of postsynaptic NMDARs, while mechanisms 3 and 4 are mediated by presynaptic NMDARs. The data presented here are most consistent with mechanisms 2 and 3 since NMDAR activation controlled presynaptic protein accumulation in the absence of glia and at synapses that lacked postsynaptic NMDARs.

NMDARs could regulate presynaptic development via four possible mechanisms (Figure 7). First, activation of postsynaptic NMDARs could induce transmission of a retrograde trans-synaptic signal sent from the postsynaptic dendrite to its presynaptic partner. Second, the NMDARs in the dendrites of a given neuron could signal transneuronally to the axon of the same neuron to control presynaptic development. Third, activation of presynaptic NMDARs by glutamate released from presynaptic terminals could be responsible for the observed effects. Fourth, glia could release glutamate that activates NMDARs on the presynaptic neuron. Our data indicate that retrograde signaling across nascent synapses is not required for NMDAR-dependent regulation of presynaptic development. Our results also demonstrate that glutamate derived from glia is not required. Therefore, our results are most consistent with a cell autonomous action of NMDARs, mediated by presynaptic NMDARs or transneuronal signaling.

Presynaptic NMDA receptors (preNMDARs) are present in the developing cerebral cortex [30,50], with the highest levels found during the peak of excitatory synapse formation [51]. These receptors are already functional at P7, the earliest age tested [51]. Therefore, preNMDARs are well-positioned to play a role in regulating excitatory synapse formation in the cerebral cortex. Although the function of preNMDARs in synaptic plasticity and short-term changes in neurotransmitter release is beginning to be understood [31], the consequences of prolonged changes in preNMDAR activity and their potential role in synapse development remain unknown. If preNMDARs facilitate accumulation of SV and AZ proteins at nascent synapses, this mechanism could ensure that functional presynaptic terminals are preferentially made and maintained, even in the absence of postsynaptic responses. This is particularly important given the prevalence of postsynaptically silent synapses during circuit development [52,53]. Interestingly, preNMDAR activation could represent a general mechanism for quality control during development of excitatory synapses since preNMDARs have been found in neurons from visual and somatosensory neocortex, entorhinal cortex, hippocampus, amygdala, cerebellum and spinal cord [30,50]. To test this new model, it will be important to develop methods for selectively activating or blocking preNMDARs for extended periods of time without affecting postsynaptic NMDARs [30].

How might activation of preNMDARs control synaptic protein accumulation? One possibility is that calcium influx via preNMDARs could enhance recruitment and stabilization of SV and AZ proteins. Cytoplasmic calcium can control trafficking of SV proteins, increasing pausing of SV protein transport at sites of synapse formation [8]. This may favor accumulation of SV proteins at nascent presynaptic terminals. It is not yet clear whether AZ protein delivery is similarly regulated by intracellular calcium.

Transneuronal signaling is also possible since recent evidence suggests that postsynaptic activity can signal to the axon, independent of action potential generation [34-38]. In cortical layer 5 pyramidal neurons (ages P13 to P19), activation of dendritic NMDARs may be responsible for cell-autonomous NMDAR-dependent facilitation of neurotransmitter release [34]. In this study, direct activation of axonal NMDARs was not observed, and it was suggested that sub-threshold postsynaptic NMDAR activity could affect presynaptic release machinery via electrical signaling. It is possible that similar mechanisms are responsible for the changes in presynaptic protein accumulation that we have observed. In the experiments described here, TTX treatment did not mimic NMDAR blockade, so the observed effects of NMDAR inhibition were probably not due to action potential-dependent depolarization. Several mechanisms for cell-autonomous control of presynaptic development by postsynaptic receptors are possible. For example, dendritic NMDAR activation could induce signal transduction that ultimately alters second messenger levels within the axon, and this second messenger could control synaptic protein accumulation. Alternatively, dendritic NMDAR-dependent signals could control the trafficking of presynaptic proteins out of the soma and into the axon.

Coincident with the period of intense synapse formation, several important aspects of visual cortical receptive fields are established, including retinotopy, ocular dominance and orientation selectivity. Activity within the visual cortex contributes to establishment of these receptive field properties [54]. NMDAR blockade interferes with development of receptive field properties in the visual system [55-57]. Therefore, control of presynaptic development by NMDARs could ultimately play a role in establishing receptive fields.

## Conclusions

Here, we have demonstrated that activation of NMDARs controls accumulation of SV and AZ proteins at developing presynaptic terminals. This regulation occurs even in the absence of retrograde signaling from postsynaptic NMDARs or glia and may also involve activation of AMPA/kainate receptors. We propose a novel model for presynaptic development in which NMDAR activation cell-autonomously regulates presynaptic terminal assembly. This could provide a feed-back mechanism capable of promoting development of active presynaptic terminals, even at silent synapses, and could contribute to activity-dependent development and refinement of cortical receptive field properties.

## Materials and methods

All studies were conducted with an approved protocol from the Case Western Reserve University Institutional Animal Care and Use Committee, in compliance with National Institutes of Health guidelines for care and use of experimental animals.

### Cell cultures and transfection

Cell culture reagents were from Invitrogen (Grand Island, NY, USA) and other chemicals were from Sigma (St. Louis, MO, USA) unless otherwise indicated. Neurons were dissociated from visual cortices of 0- to 3-day-old Long Evans rats [58] then plated (3 to 4 × 10^4 ^cells/ml) on a confluent monolayer of cortical astrocyes in Neurobasal-A medium with glutamax and B27 supplement. Astrocytes were grown on coverslips coated with collagen and poly-L-lysine and maintained in Minimum Essential Medium containing glutamax, 10% fetal calf serum, glucose (0.6%), N2 and penicillin-streptomycin. At 2 to 4 DIV, cultures were treated with anti-mitotic (5'fluoro-2'-deoxy-uridine/uridine). HEK-293 cells were maintained in DMEM with 10% fetal calf serum and penicillin-streptomycin. Neurons were transfected at 5 to 7 DIV using calcium-phosphate [59,60] or lipofectamine 2000 [58].

For growth in the absence of glia, neurons were plated directly on coverslips coated with 1 mg/ml high molecular weight poly-L-lysine. Glia were present at very low numbers in these preparations, and addition of anti-mitotic prevented their proliferation. At the time of imaging, non-neuronal cells were present at a density of approximately 3.7 × 10^12 ^mm^2^, as determined by the absence of neuronal markers, such as βIII-tubulin and tau. For these experiments, images were only collected in regions that lacked non-neuronal cells.

### Presynaptic terminal induction assay

HEK-293 cells were co-transfected with hemagglutinin-tagged neuroligin and actin-EGFP. HEK-293 cells were removed from their dishes using enzyme-free dissociation medium (Invitrogen) 12 to 24 h after transfection, washed in neuronal medium, then added to neuronal cultures (6 to 7 DIV) at 3 to 5 × 10^4 ^cells per 18 mm coverslip. APV (0.1 mM), CNQX (0.025 mM), TTX (0.001 mM) or *N*-methyl-D-aspartic acid (NMDA; 0.015 mM) were added 10 to 60 minutes later. After 24 to 48 h, cells were fixed and immunolabeled.

### Immunofluorescence and confocal imaging

At 8 to 9 DIV, cells were fixed (15 minutes, 25°C) with 4% paraformaldehyde in 0.1 M PBS containing 4% sucrose, permeabilized with 0.2% Triton X-100 in PBS, and blocked with 10% horse serum or bovine serum albumin in PBS. Antibody labeling was in 3% horse serum or bovine serum albumin for at least 1 h at 25°C or overnight at 4°C. Coverslips were mounted in fluoromount (Fisher (Waltham, MA, USA)) containing 1,4-diazabicyclo(2.2.2)octane. Primary antibodies were: mouse anti-synaptophysin (Sigma), rabbit anti-synapsin (Millipore-Chemicon (Billerica, MA, USA), Synaptic Systems (Goettingen, Germany)); rabbit anti-piccolo, rabbit anti-VGlut2, guinea pig anti-tau-1 (Synaptic Systems); guinea pig anti-VGlut1, chicken anti-GFP (Chemicon); and mouse anti-PSD95 (NeuroMab (Davis, CA, USA)). Secondary antibodies were Alexa-fluor conjugates (Invitrogen).

Imaging was performed on a Nikon C1 Plus confocal system on a Nikon Ti-E inverted microscope with 488Ar, 543HeNe and 633HeNe lasers and 40 × 1.0NA and 0.95NA objectives. Excitatory neurons were chosen for analysis based on morphology and/or expression of VGlut1. Three-dimensional image stacks were collected using EasyC1 software (Nikon) with 2 × Kalman averaging and a pixel size of 80 to 110 nm and 16 bit pixel depth. Each channel was imaged separately to avoid bleed-through. Imaging parameters were optimized to maintain fluorescence within the linear range and maximize intensity resolution. Settings were kept constant for all conditions to be compared within each experiment. Although gains were generally very similar across experiments for any given antibody label, gains were set independently for each independent experiment. Overall fluorescence was measured over 3,172 to 12,688 μm^2 ^for each field of view.

### Immunoblotting

Cultures were lysed in 1% Triton X-100 (in 50 mM Hepes, pH 7.4, 150 mM NaCl, 1 mM EDTA, protease inhibitor cocktail) on ice then incubated at room temperature in Laemmli sample buffer. Equal protein was loaded on Tris-glycine gels. Blots were probed with rabbit anti-synapsin (1:400) followed by goat anti-rabbit-horse radish peroxidase. Equal loading was confirmed by probing the same blot with MAP2 antibody. Chemiluminescence was recorded and quantified using an AlphaImager HP system (Alpha Innotech (Santa Clara, CA, USA)). All signals were below saturation and within the linear range of the detector.

### Analysis

Automated image analysis was performed using custom written macros in ImageJ. Maximum intensity Z-projections were made then background-subtracted using a rolling ball algorithm. GFP images were used to select the region of interest (axon or HEK-293 cell). Fluorescent puncta were automatically selected in the region of interest, and their intensities, areas and densities were measured. Analysis of apparent size was of images collected at 80 nm/pixel. It is worth noting that the actual sizes of AZs and clusters of SVs are near or below our spatial resolution limit; therefore, one cannot conclusively separate changes in intensity and size. Measurements were transferred to Matlab for quantification using custom-written functions. To combine the data obtained from multiple experiments, the data were normalized. For each independent experiment, data were normalized by dividing by the mean of the control values of that experiment. Then normalized values were pooled to perform statistical analysis on the entire population of presynaptic terminals. Data are presented as normalized mean ± standard error of the mean. Statistical tests were ANOVA or Wilcoxon rank sum test.

## Abbreviations

AMPA: α-amino-3-hydroxy-5-methyl-4-isoxazolepropionic acid; APV: DL-2-amino-5-phosphonopentanoic acid; AZ: active zone; CNQX: 6-cyano-7-nitroquinoxaline-2,3-dione; DIV: days *in vitro*; GFP: green fluorescent protein; NMDA: *N*-methyl-D-aspartic acid; NMDAR: NMDA receptor; preNMDAR: presynaptic NMDA receptor; PBS: phosphate-buffered saline; SV: synaptic vesicle; TTX: tetrodotoxin.

## Competing interests

The authors declare that they have no competing interests.

## Authors' contributions

MPS, CTB and SLS collected and analyzed data. SLS wrote the paper. All authors have read and approved the final manuscript.
